# Visual Working Memory Capacity Can Be Increased by Training on Distractor Filtering Efficiency

**DOI:** 10.3389/fpsyg.2017.00196

**Published:** 2017-02-17

**Authors:** Cui-Hong Li, Xu He, Yu-Juan Wang, Zhe Hu, Chun-Yan Guo

**Affiliations:** ^1^Beijing Key Laboratory of Learning and Cognition, Department of Psychology, Capital Normal UniversityBeijing, China; ^2^College of Teacher Education, Hefei Normal UniversityHefei, China; ^3^Beijing Advanced Innovation Center for Imaging Technology, Capital Normal UniversityBeijing, China

**Keywords:** visual working memory, capacity, training, filtering efficiency, CDA

## Abstract

It is generally considered that working memory (WM) capacity is limited and that WM capacity affects cognitive processes. Distractor filtering efficiency has been suggested to be an important factor in determining the visual working memory (VWM) capacity of individuals. In the present study, we investigated whether training in visual filtering efficiency (FE) could improve VWM capacity, as measured by performance on the change detection task (CDT) and changes of contralateral delay activity (CDA) (contralateral delay activity) of different conditions, and evaluated the transfer effect of visual FE training on verbal WM and fluid intelligence, as indexed by performance on the verbal WM span task and Raven’s Standard Progressive Matrices (RSPM) test, respectively. Participants were divided into high- and low-capacity groups based on their performance in a CDT designed to test VWM capacity, and then the low-capacity individuals received 20 days of FE training. The training significantly improved the group’s performance in the CDT, and their CDA models of different conditions became more similar with high capacity group, and the effect generalized to improve verbal WM span. These gains were maintained at a 3-month follow-up test. Participants’ RSPM scores were not changed by the training. These findings support the notion that WM capacity is determined, at least in part, by distractor FE and can be enhanced through training.

## Introduction

Working memory (WM) is a critical cognitive processing system that retains and manipulates limited amounts of information simultaneously within short periods of time (Baddeley, 1992, 2003). There are marked individual difference in WM that may limit the efficiency of higher-order cognitive processes, such as language comprehension, thought, reasoning, and problem solving (Alloway et al., 2006; Tillman et al., 2008; Carretti et al., 2009). However, the nature of such individual differences is unclear. visual working memory (VWM) is one of several domains of WM. Filtering efficiency (FE) is the ability to exclude irrelevant information from accessing VWM. Vogel et al. (2005) found that differences in FE may account for performance on VWM tasks.

Vogel et al. (2005) developed a task to reliably distinguish between high and low VWM capacity (see Change Detection Task). The task measures the amplitude of contralateral delay activity (CDA) as a neural index of the number of retained items. CDA is the negative-going voltage in the hemisphere contralateral to a memorized hemifield that is larger than the voltage change observed in the ipsilateral hemisphere during the intra-trial delay period (Vogel and Machizawa, 2004). CDA amplitude increases with memory-set size, and reaches an asymptote at VWM capacity. When presented with two or four memory items (in the absence of distractors), low- and high-capacity groups show similar CDA amplitudes. However, in the presence of distractor items (two target items along with two distractors), the CDA amplitude of the high-capacity group is equal to that observed when presented with two target items alone, while the CDA amplitude of the low-capacity group resembles the amplitudes recorded when presented with four target items (Vogel et al., 2005). That is, it appears that the so-called low-capacity group maintained at least as much information as the high-capacity group, but displayed lower FE.

Since the study by Vogel et al. (2005), the relationship between VWM capacity and attention selection has been extensively explored. Luria et al. (2016) highlighted important findings demonstrating that attentional control, which prevents distractors from gaining access to VWM, rather than the maximum storage capacity limit, determines individual differences in VWM and CDA. Melcher and Piazza (2011) proposed the idea that attentional priority and saliency of items influence the availability of VWM resources and affects VWM capacity. Similarly, Pedale and Santangelo (2015) found that a bottom-up sensory saliency modulation effects the contents of VWM, which also affects the overall amount of information successfully recollected. In sum, the above research provides powerful evidence for a link between attention selection and WM performance.

Prior research has suggested that WM capacity is limited but not fixed. WM capacity can be improved by training protocols (Holmes et al., 2009; Van der Molen et al., 2010; Owens et al., 2013). For instance, Borella et al. (2010) demonstrated that older adults could benefit from a verbal WM training program. Similarly, Carretti et al. (2013) found that practice could improve performance in a verbal WM task in amnesic patients with mild cognitive impairment. To date, the majority of studies examining training effects on WM performance have focused on cognitively impaired patients and thus, it is unclear whether similar improvement can be achieved in a cognitively normal population. The current study investigated whether the WM capacity of low-capacity individuals can be improved by training aimed to facilitate FE.

Transfer of acquired skills is probably the ultimate goal of training study. Several recent studies have demonstrated that training that focusing on one type of WM leads to improved performance on other not-practiced tasks, including VWM, fluid intelligence, and long-term memory (Carretti et al., 2013). For example, practice with the N-back short-term memory task has been shown to enhance performance in a reasoning ability test (Zhao et al., 2011). In the present study, we employed two tests to examine the transfer of the benefits of FE training to other tasks including VWM and fluid intelligence.

Prior studies have shown that WM capacity is related to fluid intelligence (i.e., knowledge-independent problem solving and reasoning) (Kyllonen and Christal, 1990). For instance, Unsworth et al. (2015) found that individual differences in WM delay activity (CDA) was significantly correlated with cognitive abilities such as general fluid abilities and this association was in part due to differences in attention control. Klingberg et al. (2002) reported that WM training significantly improved fluid intelligence performance in children with attention deficit hyperactivity disorder (ADHD). However, results from studies on the transfer of the benefits of training to other tests of intelligence have been inconsistent, and some studies have reported no changes in intelligence with training (Morrison and Chein, 2011). Therefore, the extent to which WM training can affect fluid intelligence remains unclear.

Baddeley and Hitch’s (1974) WM model postulates that the visuospatial sketchpad and the phonological loop are modality-based temporary stores for visual and verbal acoustic material, respectively. To date, relatively few studies have explored the relationship between these two modalities. One study found no interaction between verbal and visual WM (Cocchini et al., 2002). However, Morey and Cowan (2005), using a dual task paradigm, found that interference between verbal and visual WM occurred when there was explicit retrieval of verbal load during the maintenance period of a visual task, or when large silent verbal or visual loads were held at the same time. Thus, we hypothesized that if visual and verbal WM interact, then visual WM training would also influence performance on verbal WM tasks.

Given that inefficient stimulus selection may result in poor WM performance, and that WM capacity can be improved through training, the present study investigated whether VWM capacity can be improved in low-capacity individuals through training with adaptive tasks aimed at improving neural filtering. In addition, whether such training could bring VWM performance and CDA to the same level as a high-capacity group was also investigated. Additionally, we tested whether gains in VWM can transfer to other WM systems, namely verbal WM, and whether the improvement affects performance on a fluid intelligence test. Finally, we tested whether improvements in performance were maintained long term (3 months).

## Materials and Methods

### Subjects

Thirty-eight right-handed college students (mean age, 21.4 years, 13 males) from Capital Normal University were recruited to participate in the pre-training experiment between September 2013 and April 2014. All participants included in our analysis had normal or corrected-to-normal vision and passed the Ishihara test for color blindness. Before participating in the experiments, all gave written informed consent, and were compensated for their time. Our protocol was reviewed and approved by the ethics committee of Capital Normal University.

### General Training Procedure

The study was composed of four parts: a pre-training, training, a post-training, and a follow-up assessment. We divided the participants into high- and low-capacity (of WM) individuals using a median split of their pre-training results. Then, only the low-capacity individuals took part in FE training. WM capacity was assessed immediately after training in the post-training and at a follow-up assessment 3 months later.

**FIGURE 1:**
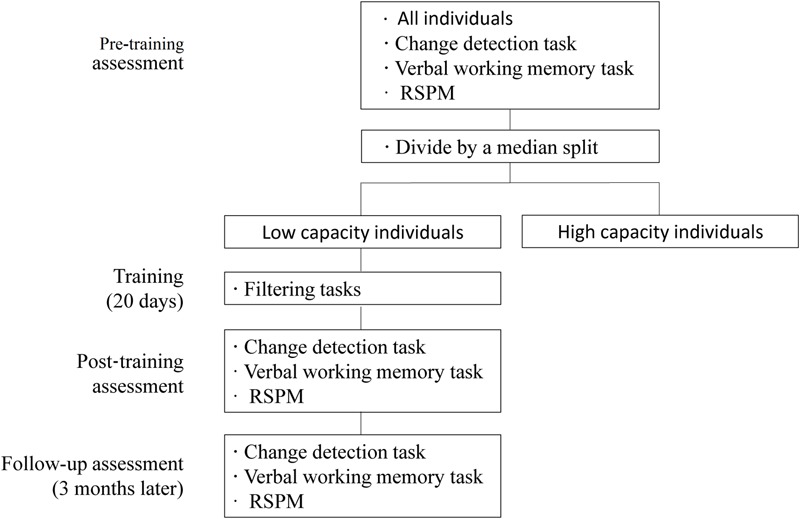
**General training procedure**.

At the pre-training assessment, all participants completed a VWM task, a verbal WM span task, and the RSPM (Raven standard progressive matrices) test to estimate their VWM capacity (when presented with four items), verbal WM capacity, and fluid intelligence before training. FE training was given only to those participants with sub-median performance on the VWM pre-training assessment, that is, only to the low capacity group. At the post-training assessment, immediately after the training period, we re-assessed the VWM capacity of the participants who had completed FE training, and investigated if there were transfer effects to verbal WM and/or fluid intelligence. All tests administered at the pre- and post-training time points were re-administered again at a follow-up time point (3 months after training). Parallel versions of the WM tasks (with different stimuli) were used and balanced across the assessment time points (**Figure 1**).

Training occurred in a fixed computer room and involved 20 daily sessions (5 days per week) of 50 min each day. The training schedule (i.e., hours in which particular participants completed their sessions) was determined by the participants based on their performance.

### Tasks

#### Change Detection Task (CDT)

The change detection task (CDT) was used to measure VWM capacity as described by Vogel et al. (2005) and served as the main task of the experiment. Visual stimuli were displayed on a gray background (8.2 cd m^-2^) on a 15-inch CRT monitor at a viewing distance of 70 cm. The participants were seated in an electrically isolated, soundproof room with dimmed lighting, and were required to maintain fixation at the center of the screen throughout the trial.

Stimuli were presented within two rectangular regions (4° × 7.3°) with their centers 3° to the left and right of a central fixation cross. Stimulus arrays consisted of two or four colored rectangles (1.21° × 0.64°) whose orientations were selected randomly from four possible values (horizontal, vertical, left 45°, right 45°) in each hemifield. Stimulus positions were randomized across trials with the only constraint being that the distance between any two rectangles within a hemifield was 2° (center-to-center). All pictures were matched for luminance.

**FIGURE 2:**
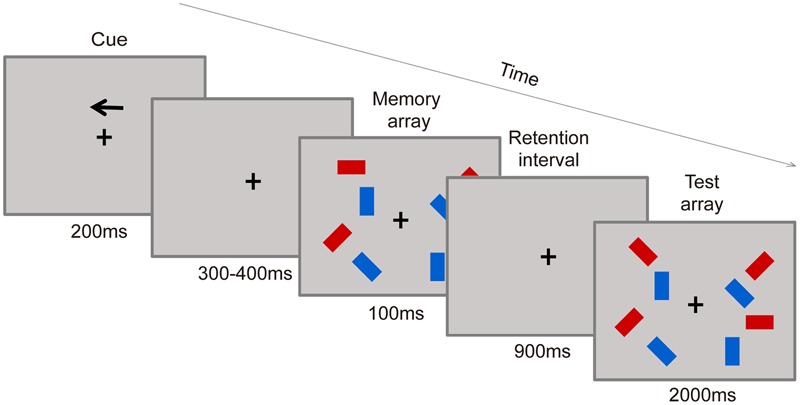
**Visual working memory (VWM) task procedure.** A filtering condition example is shown.

Participants were asked to judge whether the orientations of the rectangles in the target hemifield had changed or not. As shown in **Figure 2**, each trial consisted of a study phase, retention phase, and test phase. Each trial began with the presentation of an arrow-shaped cue that pointed the left or right hemifield in which the items needed to be maintained above the fixation point for 200 ms, and this cue was followed by a variable delay (range, 300–400 ms). Then, the memory array appeared for 100 ms, followed by a 900-ms blank interval. Finally, the test array was displayed for 2000 ms. Participants were asked to report whether the rectangle in the test display changed orientation or not from the sample displayed at the same position by pressing one of two response keys. The mapping between the buttons and responses was counterbalanced across participants. The test display rectangles were identical to the sample displays in half of the trials. In the other half, a single red rectangle in the target hemifield was changed compared to the sample displays. The goal of accuracy over speed was stressed in the instructions.

The sample displays contained three conditions: two-item, four-item, and filtering. For the two-item and four-item conditions, there were two or four red rectangles within each hemifield, respectively. And in the filtering condition, there were two red rectangles presented along with two blue rectangles in each hemifield, with only the red rectangles needing to be maintained. Display conditions (two-item, four-item, and filtering items), arrow direction (left, right), change (yes or no) were randomized within each block. Each participant performed 12 experimental blocks of 48 trials, and a practice session of 20 trials was administrated before the experimental blocks. Within each block, participants were given a 10-s break after half of the trials were completed. The experiment lasted approximately 70 min.

#### Transfer Tasks

##### Verbal working memory span task (VeWMST)

The VeWMST was compiled from the operation word span task developed by La Pointe and Engle (1990). This task is based on the process-storage function models of WM. Participants were presented with a series of sum-word pairs on the center of the screen, such as “(8÷4) + 5 = 8? appearance”, “(2 × 7) – 6 = 7? tire”. Sum calculation (all answers were <20) is considered processing while maintaining the words is considered storage. The participants were required to read aloud what they saw on the screen first, then judged whether the answer in the math sentence shown was correct, and gave their response by pressing one of the two response keys. At the same time, they were supposed to keep the word following the sum in mind. After several pairs were presented, “???” would appear at the moment the participants were required to write down the words in the group as in order as possible within 1 min. Half of the sums’ answers were correct, and the response button was counterbalanced across participants. The sums and words used in the pre-training, post-training, and follow-up assessment were different but parallel.

Each series consisted of two to six sum-word pairs, the number of pairs presented in sequence was: 2, 2, 2, 3, 3, 3, 4, 4, 4, 5, 5, 5, 6, 6, 6. In total, there were 15 series and 60 pairs. When a participant recalled a word correctly, he would get a point. The scores were considered to be valid only if the accuracy of the sums reached 80%. The final score corresponded to the total number of trials with correct recalls (maximum score = 60).

##### Raven’s standard progressive matrices (RSPM) test

The RSPM test was used to estimate the fluid intelligence of the participants. Items consisted of abstract shapes and curved lines, with some part of each item missing. The goal is to identify the right component to fill in the missing part with from six or eight small pictures below (chosen by pressing the keys) without a time constraint. The test was scored in the same way as the VeWMST (maximum score = 60).

### Training

**FIGURE 3:**
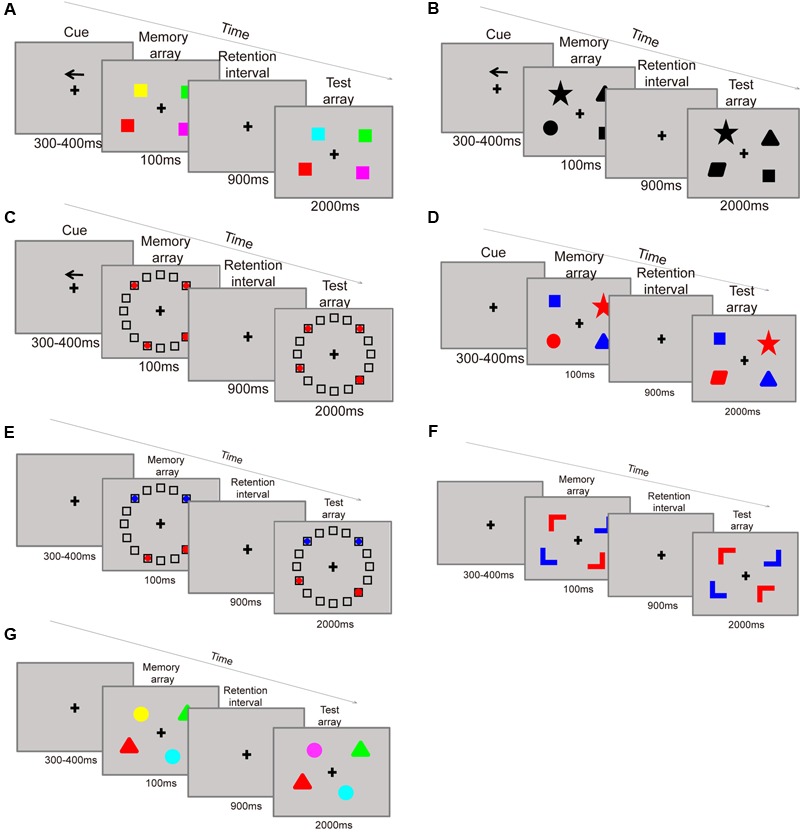
**Training paradigms: remember the color on the cue side **(A)**.** Remember the shape on the cue side **(B)**. Remember the location of items on the cue side **(C)**. Remember the shape of the red items **(D)**. Remember the location of the red items **(E)**. Remember the color of triangles **(G)**.

The training consisted of seven kinds of change detection paradigms, all administered in a filtering condition. In the paradigm, each item contains two attributes: a target attribute and a cue attribute. Participants were instructed to focus on the target information in the presence of irrelevant (distractor) information in the display set. Participants were asked to remember categories of cued stimuli as: (paradigm 1) colors on the cued side (**Figure 3A**), (paradigm 2) shapes on the cued side (**Figure 3B**), (paradigm 3) item locations on the cued side (**Figure 3C**), (paradigm 4) shapes of target (red-colored) items (**Figure 3D**), (paradigm 5) locations of target (red-colored) items (**Figure 3E**), (paradigm 6) orientations of target (red-colored) items (**Figure 3F**), and (paradigm 7) colors of target (triangle-shaped) items (**Figure 3G**). Specifically, in paradigms 1, 2, and 3, participants are asked to filter the items located in the opposite direction of the arrow cue. For example, the items in the paradigm of remember colors on the cued sided (**Figure 3A**) contains two attributes of shape and location (in the left or right side), shape is the target attribute, and location is used as cue. Firstly, the participant should remember the direction of the arrow, if the arrow pointed to left side, then the participant need only to remember the color of the square on the left side. For participants in this conditions the filtering distractors are the color of the squares on the right side. On the contrary, if the cue pointed to right then remembering the color of the squares on the right side and suppress the left ones are required. While in the paradigms 4, 5, and 6, participants are asked to filter the blue items on both sides of the screen. For example, remember shapes of target (red-colored) items (**Figure 3D**) needs participants to remember the shapes of the red squares, while ignoring the blue ones. Thus, shape is the target attribute and color is the cue. At the test phase, the participants only need to judge whether the shapes of the red squares have changed or not. The last paradigm 7 is remember colors of target (triangle-shaped) items based on its shape (**Figure 3G**). In this paradigm, the targets are the color of triangle items, and the distractors are the color of round items. For this paradigm the participants need to report the color of the triangles, requiring them to maintain the object’s representation in their memory until it is time to provide a response. To maximize the training effects, the trials became more difficult over the training period of each category by successively increasing set sizes which ranged from two to four items.

Each participant completed two different categories of tasks which included 336 trials of each task 1 day in about 50 min. An automatic adaptive training procedure was utilized to increase the difficulty within categories. The initial trials were of low difficulty and became increasingly more difficult for each category once the participants reached 90% accuracy. For example, a participant who reaches 90% accuracy on paradigm A, but not for paradigm B will have the difficulty of the test items increase for paradigm A but not for paradigm B. Thus, with different memory capacities, each participant has his own training schedule depending on his scores the day before.

### Data Analyses

#### Behavioral Data

Memory performance was indexed by accuracy (the traditional index of memory capacity) as well as Cowan’s *K*-value (Cowan, 2001), calculated according to the following formula:

K=S(H−F)

Where *K* is memory capacity, *S* is array size, *H* is hit rate, and *F* is false alarm rate.

As has been done previously (Lee et al., 2010), we used the *K*-value in the four-item condition to estimate each participant’s memory capacity. We used unnecessary storage (US) index to estimate filtering ability directly (Lee et al., 2010), according the following formula:

US=Ktwo items−Kfiltering items

#### Electroencephalography (EEG)

Event-related potentials (ERPs) were recorded from 62 scalp sites with a Neuroscan system via Ag/AgCI electrodes embedded in an elastic cap. All sites were recorded with a left-mastoid reference and re-referenced offline to the average of the left and right mastoids. Horizontal electrooculogram (EOG) was recorded bipolarly from two electrodes placed approximately 1 cm to the left and right of the outer canthi of the eyes. Vertical EOG was recorded from electrodes placed above and below the left eye. Impedances were kept below 5 kΩ. Band-pass of the amplifier system was set to 0.05–100 Hz, and the signals were digitized with 500 Hz. The ERPs were segmented into 1300-ms epochs starting from 200 ms before the onset of the memory display and encompassing the whole retention interval, and then subjected to baseline correction with the 200-ms window preceding the memory display. Trials with EEG voltages exceeding ±75 μv were excluded from the analysis and epochs containing blinks or eye movements (>1°) were excluded from further analysis.

Data from five pairs of electrodes (O1/2, PO3/4, PO7/8, P5/6, and P7/8) at posterior parietal and occipital areas were subjected to CDA analysis (Vogel and Machizawa, 2004; McCollough et al., 2007). Difference waves were computed by subtracting ipsilateral waves, averaged from the electrodes ipsilateral to the memory side, from contralateral waves, averaged from the electrodes contralateral to the memory side.

To estimate filtering ability from ERPs, FE was calculated according the following formula:

FE=(F−D)/(F−T)

Where *F* is CDA amplitude induced by four items, *D* is CDA amplitude for the filtering (distractor) condition, and *T* is CDA amplitude induced by two items.

## Results

### Pre-training

Based on the initial behavioral analysis, 19 participants with CDT performance above the median (*K* = 2.45) constituted the high capacity group while the 19 with performance below the median constituted the low capacity group. Three of the participants in each group were excluded from the ERP data analyses, leaving group sizes of 16, for the following reason: >30% trials rejected for excessive eye movement (one female and two males in the low capacity group, and three male in the high capacity group).

#### Behavior

**FIGURE 4:**
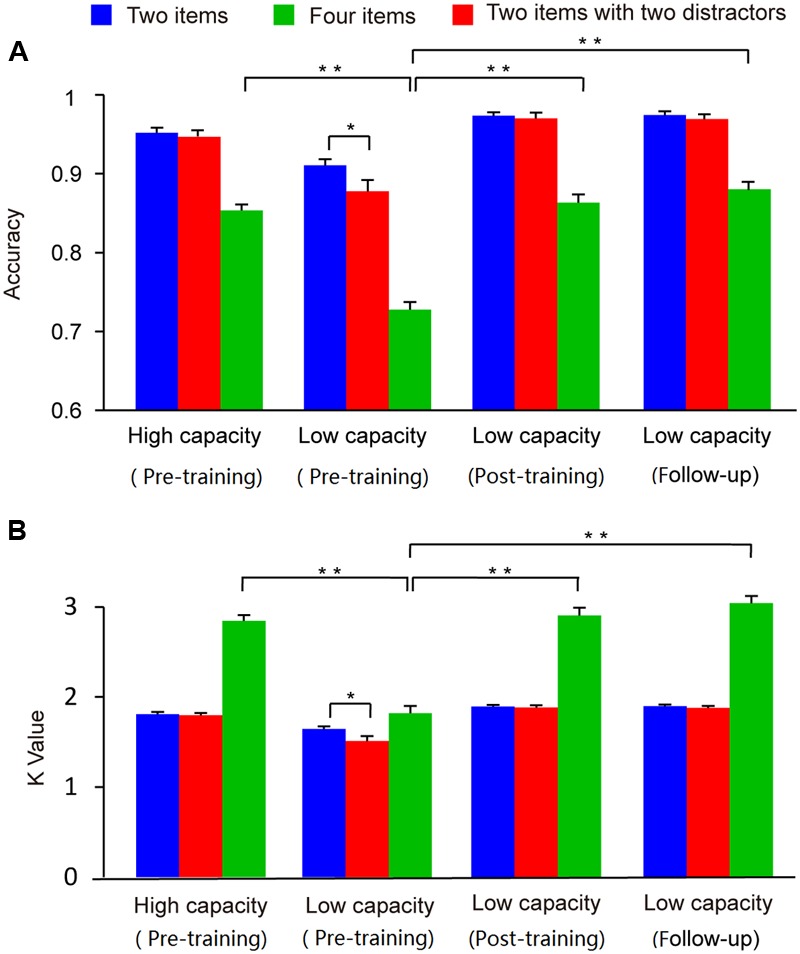
**Mean accuracy (A)** and Cowan K-values **(B)** for high and low capacity groups at the pre-training, post-training, and follow-up assessment. Asterisks indicate statistical differences at ^∗^*p* < 0.05 or ^∗∗^*p* < 0.01.

The accuracy and Cowan *K* data for the two groups across the three conditions (two-item, four-item, and filtering) are presented in **Figure 4**. In order to compare with the previous studies directly (Vogel et al., 2005), and the patterns of the results regarding group differences were similar with accuracy and *K*-values, we used only the *K*-values as the behavior index in our subsequent analysis. A two-way ANOVA of Cowan’s *K* with condition (two-item vs. four-item vs. filtering) as a within-subject factor and group (high capacity vs. low capacity) as a between-subject factor revealed main effects of condition (*F*_2,72_ = 159.49, *p* < 0.001) and group (*F*_1,36_ = 81.76, *p* < 0.001). As assumptions of sphericity were violated, Greenhouse-Geisser corrected values are reported here. There was a significant interaction between condition and group (*F*_2,72_ = 61.90, *p* < 0.001). Confirming that the grouping was appropriate, the WM capacity of high capacity group in the four-item condition was significantly better than that of low capacity group (*t*_36_ = 10.09, *p* < 0.001).

**FIGURE 5:**
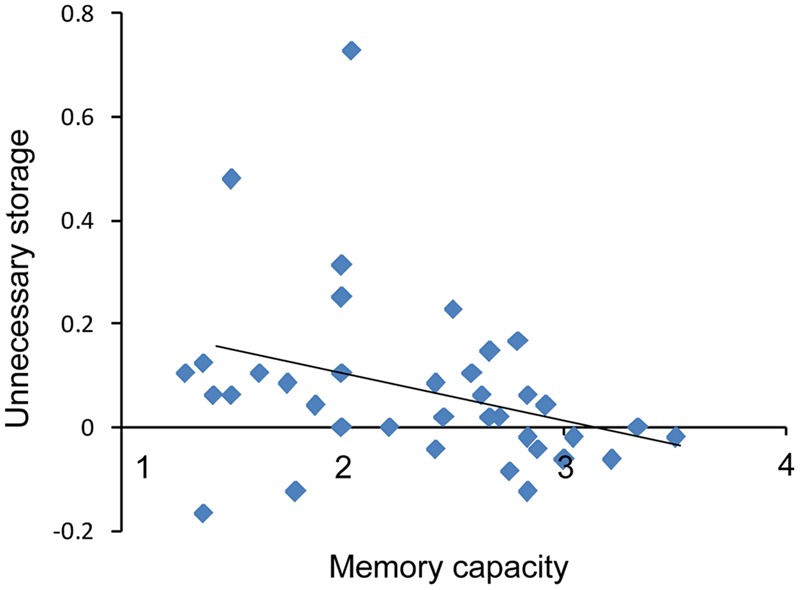
**Inverse correlation between individuals’ memory capacity scores and US**.

**FIGURE 6:**
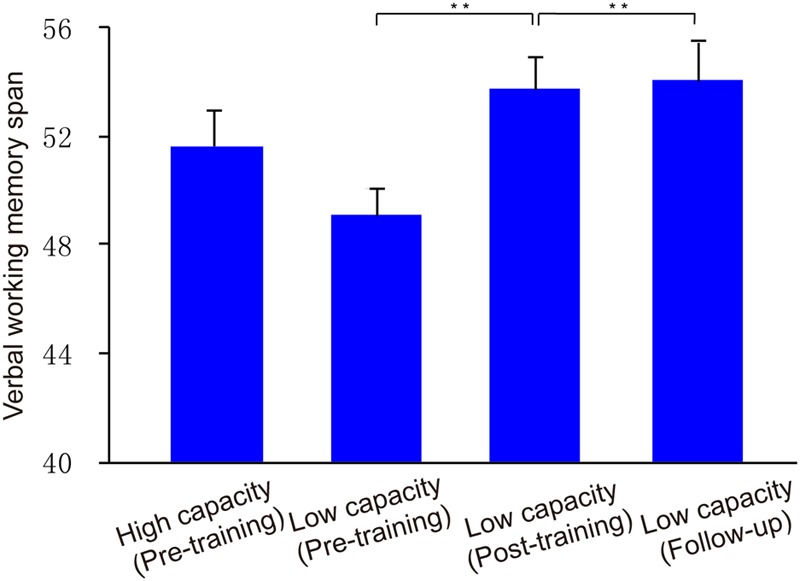
**Verbal WM performance.** Group VeWMST scores at pre-training, post-training, and follow-up assessments. Asterisks indicate statistical differences at ^∗∗^*p* < 0.01.

To investigate ability to filter distractor items, we compared capacities under the filtering versus two-item condition. *Post hoc* comparisons showed that *K*-values in the filtering condition were lower than those in the two-item condition for the low capacity group (*t*_18_ = 2.74, *p* < 0.05), whereas the *K*-values obtained in these two conditions were similar to each other for high capacity group (*t*_18_ = 0.70, *p* > 0.05). That is, the low capacity group retained more irrelative information when presented with distractors. As shown in **Figure 5**, there is a marginal significant negative correlation between US and memory capacity [*r* = -0.32, *p* = 0.05]. Moreover, US was lower for the high capacity group than for the low capacity group (*t*_36_ = 2.35, *p* < 0.05). At the pre-training, neither VeWMST (*t*_36_ = -1.55, *p* > 0.05) nor RSPM test performance (*t*_36_ = -0.38, *p* > 0.05) differed significantly between the high and low capacity groups (**Figure 6**, respectively). No correlation was found between VeWMST and *K*-values (*r* = 0.20, *p* = 0.24).

#### Electroencephalography (EEG)

**FIGURE 7:**
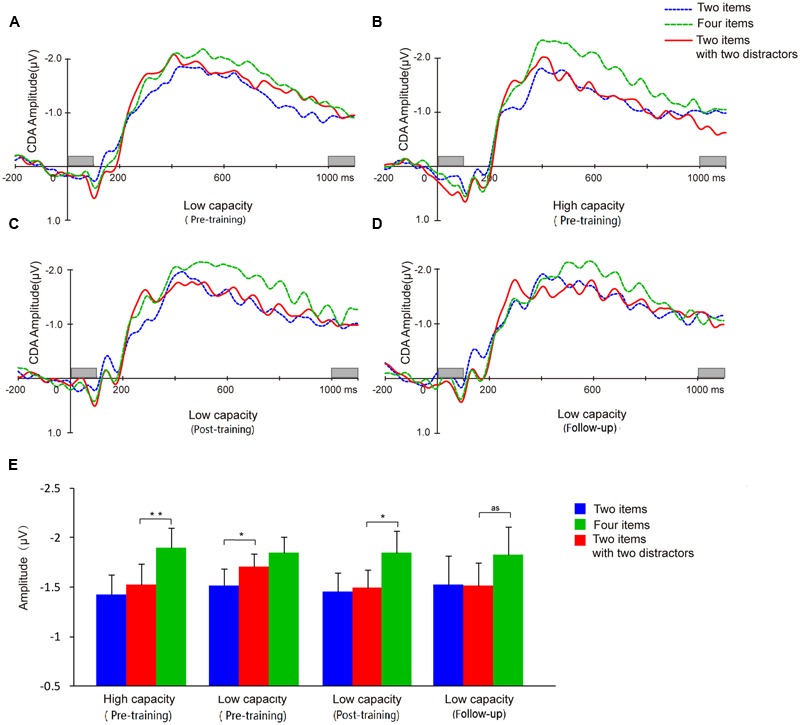
**Contralateral delay activity (CDA) waveforms and CDA amplitude means.** Grand average ERP difference waves for the low capacity group at pretest **(A)**, posttest **(C)**, and follow-up **(D)**. **(B)** Grand average ERP difference waves of high capacity individuals at pretest. **(E)** CDA amplitudes from each of the three conditions for each group at pre-training, post-training, and follow-up. And as means approach significant. Asterisks indicate statistical differences at ^∗^*p* < 0.05 or ^∗∗^*p* < 0.01.

Difference waveforms from the three conditions (two-item, four-item, filtering) collapsed across five electrodes began to separate between the high and low capacity groups after the stimulus set had appeared for 200 ms (**Figure 7**). Mean CDA amplitudes (300–900 ms after memory array onset) are compared across experimental time points in **Figure 7E**.

A two-way ANOVA of mean CDA amplitude was conducted with condition (two-item vs. four-item vs. filtering) and group (high capacity vs. low capacity) as factors. As assumptions of sphericity were violated, Greenhouse-Geisser corrected values are reported. The results revealed a main effect of condition (*F*_2,60_ = 17.11, *p* < 0.001), but not group (*F*_2,60_ = 1.91, *p* > 0.05), and no condition × group interaction (*F* < 1). CDA amplitude was higher in the four-item condition than in the two-item condition (high capacity group, *t*_15_ = 4.76, *p* < 0.001; low capacity group, *t*_15_ = 2.41, *p* < 0.05), confirming that CDA amplitude is sensitive the number of items presented. To compare with the results of Vogel et al. (2005), we used the same way in their paper to analysis our data below.

A dependent *t*-test for the CDA amplitude of the high and low capacity groups indicated that the WM resources consumed when subjects were retaining four items did not differ between the two groups (*t*_30_ = 0.02, *p* > 0.05). That is, although the capacity estimated by the behavior index was lower for the low capacity group, the information retained when presented with four items was the same as that retained by the high capacity group.

Contralateral delay activity amplitudes were compared across conditions to assess filtering ability. For the high capacity group, CDA amplitude in the filtering condition was similar to the CDA amplitude in the two-item condition (*t*_15_ = 1.21, *p* > 0.05), but significantly smaller than that in the four-item condition (*t*_15_ = 4.84, *p* < 0.001). By contrast, for low capacity group, CDA amplitude in the filtering condition was similar to the amplitude in the four-item condition (*t*_15_ = 1.19, *p* > 0.05), but significantly larger than that in the two-item condition (*t*_15_ = 2.21, *p* < 0.05).

**FIGURE 8:**
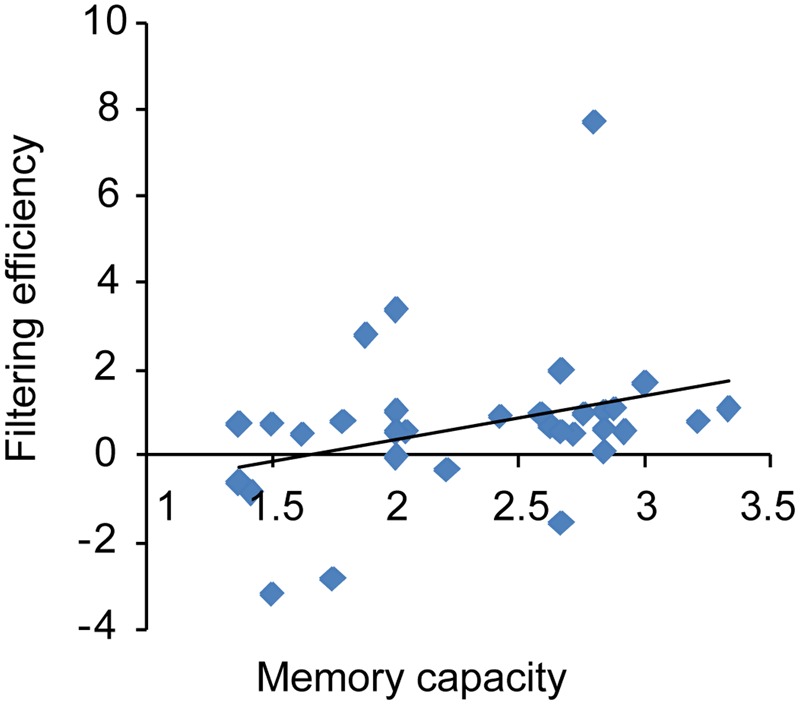
**Correlation between an individual’s memory capacity and filtering efficiency**.

As shown in **Figure 8**, WM capacity (Cowan’s *K*) correlated marginally with FE (*r* = 0.32; *p* = 0.07), indicating that high WM capacity was associated with FE in a VWM paradigm. No correlation was found between VeWMST and FE (*r* = 0.04, *p* = 0.82).

### Post-training

Two participants in each group did not complete all of the post-training tasks. Therefore the post-training results reflect the data on the remaining 14 participants per group for both behavior and EEG experiments.

#### Behavior

Training increased WM capacity, as reflected by CDT performance, in the low capacity group (*t*_13_ = 11.94, *p* < 0.001 pre-training *vs*. post-training), and their post-training WM capacity was similar to that observed for the high capacity group in the pre-training phase (*t*_31_ = -0.56, *p* > 0.05). With respect to the effect of the training on filtering ability, we found that the low capacity group’s post-training WM capacity in the filtering condition was similar to that observed in the two-item condition, rather than that observed in the four-item condition (vs. two-item condition, *t*_13_ = 0.57, *p* > 0.05; vs. four-item condition, *t*_13_ = 13.60, *p* < 0.001). We compared US of the low capacity group between the pre- and post-training time points, and found that their post-training US showed a trend toward being reduced relative to their pre-training US (*t*_13_ = 2.15, *p* = 0.051). We used the difference between the capacities of the post- and pre-training time points as the index of training gains, and found capacity was marginally correlated with gains of training (*r* = -0.51; *p* = 0.065). Indicating that individuals with lower pre-training capacity have a greater benefit than those with high pre-training capacity.

With respect to transfer effects, we observed a positive effect of training on VeWMST performance (*t*_13_ = 4.03, *p* < 0.001 vs. pre-training), but no change in RSPM scores (*t*_13_ = 0.40, *p* > 0.05 vs. pre-training). There was no correlation between VeWMST and *K*-value (*r* = -0.26, *p* = 0.38).

Intra-individual test–retest reliability was assessed using the Pearson Correlation Coefficient, which was 0.50 for *K*-values (*p* = 0.072).

RTM (regression to mean) effect is the phenomenon that if the value measured in the first time is extreme, it will be regressed to the average value. It was calculated by a method proposed by Barnett et al. (2005, 2015). The analysis observed an increase of 1.04 (95% CI: 0.85, 1.23), which is greater than our estimated RTM effect of 0.41 for our low capacity group. Which we interpret as a real change for the low capacity group.

#### Electroencephalography (EEG)

Contralateral delay activity amplitude was sensitive to the number of the items held in memory (*t*_13_ = 2.26, *p* < 0.05). In the post-training phase, the low capacity group’s CDA amplitude in the filtering did not differ from that in the two-item condition (*t*_13_ = 0.30, *p* > 0.05), but was significantly lower than that in the four-item condition (*t*_13_ = 2.95, *p* < 0.05), demonstrating that an improvement in FE had been achieved.

Different from the results of behavior, the low capacity group’s CDA in either two or four items condition has not improved (*t*_13_ = 0.05, *p* > 0.05; *t*_13_ = -0.35, *p* > 0.05). There was no correlation between VeWMST and CDA (*r* = -0.22, *p* = 0.44).

Intra-individual test–retest reliability was assessed using the Pearson Correlation Coefficient, which was 0.64 for *K*-values (*p* = 0.01).

### Follow-Up

All of the participants who took the post-training assessments were re-examined in the follow-up phase of the study.

#### Behavior

A one-way ANOVA of Cowan’s *K*-value conducted with time point (pre-training, post-training, and follow-up) as a within-subjects factor revealed a main effect of time point on WM capacity (*F*_2,26_ = 74.91, *p* < 0.001). *Post hoc* comparisons showed that WM capacity at the follow-up assessment was significantly higher than that at the pre-training (*t*_13_ = 9.99, *p* < 0.001), but not different from WM capacity at the post-training assessment (*t*_13_ = 1.52, *p* > 0.05).

Similar to our post-training observations, the low capacity group’s WM performance in the filtering condition was similar to that in the two-item condition (*t*_13_ = 1.49, *p* > 0.05). Likewise their US remained similar to that seen in the post-training phase (*t*_13_ = 0.56, *p* > 0.05), indicating that they were able to keep the relevant items in memory selectively at follow-up. With respect to transfer effects, the low capacity group’s VeWMST scores at follow-up were significantly greater than at the pre-training assessment (*t*_13_ = 4.03, *p* < 0.001), and similar to their post-training VeWMST scores (*t*_13_ = 0.27, *p* > 0.05).

#### Electroencephalography (EEG)

Similar to our observations at earlier time points, we found that CDA amplitude remained sensitive to the number of items in the task (i.e., two-item vs. four-item) in the follow-up assessment (*t*_13_ = 2.64, *p* < 0.05). Meanwhile, the CDA amplitude observed in the filtering condition showed a trend toward being smaller than that in the four-item condition (*t*_13_ = 2.09, *p* = 0.057), but did not differ from that in the two-item condition (*t*_13_ = 0.07, *p* > 0.05). The follow-up results demonstrated maintenance of the effects of training on FE for 3 months.

## Discussion

In the present study, training lead to significant improvement in FE in a low VWM capacity group, such that VWM capacity of the low-capacity group did not significantly differ from the high VWM capacity group. In addition, the benefits of training transferred to a verbal WM task, and were maintained for at least3 months. Intelligence, as reflected by RSPM scores, was not affected by training. Although there was no training control group in the present study (limitations and explanations will be discussed below), these results indicate that WM can be improved by training, in agreement with previous studies (Holmes et al., 2009; Van der Molen et al., 2010; Owens et al., 2013).

Filtering efficiency was the core component of training in the current study and as such, FE was analyzed at different stages of training. During the pre-training assessment, it was necessary for subjects to maintain the orientation of the two target (red) items, when presented either alone or with filtering information. The low-capacity group performed poorer when the filters were present indicating that low-capacity individuals retain some information about the distractors (filters) which alters their ability to detect the targets. However, the performance of the high-capacity group did not change when distractors were present, indicating that only low-capacity individuals have a filtering deficit. It follows, therefore, that filtering training should have a greater influence on low-capacity individuals than high-capacity individuals. To maximize the observation of the training effect, only low capacity individuals were included in our sample. In fact, there was a positive correlation between capacity gain and filtering training. The participants who showed the greatest increase in capacity following filtering training were those who performed at the lower end of the pre-training assessment, while individuals who performed at the higher end at the pre-training assessment showed only moderate improvement in capacity scores following filtering training. At the post training assessment and at a 3-month follow-up, low-capacity individuals performed equally well at both the two-item condition and the filtering condition. These results indicate that training improved efficiency for low-capacity individuals. There was a trend toward a negative correlation between VWM capacity and US, which measures FE directly as well as the effect of distractor information on memory. These result indicate that higher-capacity individuals have lower US, as these individuals maintain lower amounts of distractor information and have higher FE compared to low-capacity individuals.

Given that CDA amplitude is a sensitive neurophysiological index of the amount of information stored in VWM, which excludes distractor information from encoding and test phase in the behavior results (Ikkai et al., 2010), the present CDA results provide evidence that WM is neurophysiologically plastic. Consistent with the work of Vogel et al. (2005), there was a positive correlation between VWM capacity and FE in the current study and in addition, both measures were positively affected by training. These results replicate the important proof of concept that differential allocation of memory capacity to distractors may underlie individual differences in WM capacity.

After 20 days of training, the CDA and *K*-values of the low-capacity group did not significantly differ from the high-capacity group. That is, with training, the low-capacity group grew more capable of excluding irrelevant items, and thereby physiologically and behaviorally were similar to the high-capacity group. Moreover, the improvements were maintained at a 3-month follow-up assessment. These results not only demonstrate that training can enhance WM ability, but also provide support for the hypothesis that individual differences in WM capacity may be the result of differences in ability to allocate memory capacity resources selectively.

The design of the present study was unique in that the training intervention focused on filtering ability rather than the capacity of WM. The current paradigm strongly verifies Vogel et al.’s (2005) findings that low WM capacity can be at least in part, attributed to an undeveloped filtering ability and that filtering ability can be enhanced through training. Indeed, a growing number of studies have revealed possible overlapping constructs between attention and WM, and selective attention influences WM performance. Top-down modulation may be the common neural mechanism in the ability to focus attention on task-relevant stimuli while ignoring irrelevant distractions (Gazzaley and Nobre, 2012). This effect engages during the encoding, maintenance and retrieval phase of WM. Moreover, recent studies have shown that selective attention not only enhances WM performance, but also contributes to determining WM contents (Melcher and Piazza, 2011). The current findings further revealed the important function of early attentional selection on WM performance. High-capacity and low-capacity individuals may maintain the same amount of information in memory, while displaying differences in memory task performance due to FE. These findings may indicate that the index of capacity, *K*, may not reflect the actual capacity of an individual.

During the training phase, we used a change detection paradigm that included various filtering conditions, with set sizes ranging from two to four items. The results indicated that the training may have affected both filtering ability and memory storage. However, as the pure index of the number of retained items, the CDA amplitude in response to two and four items did not change between pre-training assessment and post-training. Taken together, these results suggest that the number of items stored in memory have not been increased as a result of training. Combined with the FE results, we observed no effect of memory storage training on task performance at post-training or follow up stages. The only training to affect performance was filtering training, which improved capacity for low-capacity individuals. It has been suggested that neural mechanisms underlying the filtering process are associated with a neuronal gatekeeper network which includes the basal ganglia (BG) and prefrontal cortex (pFC) and is believed to play a critical role in the access to WM storage (McNab and Klingberg, 2008). In addition, the current results indicate that filtering training rendered WM more efficient by strengthening the neuronal gatekeeper network, and thus improved the ability of inhibiting irrelevant information from being unnecessarily stored in memory (Schmicker et al., 2016).

In the present study, the change-detection task was used such that results could be directly compared to those of Vogel et al. (2005). However, one established limitation of the change-detection approach is that when array size exceeds WM capacity, sensitivity is reduced (Gold et al., 2007). In the present experiment, when the set size was large (four items), participants performed significantly higher (79.04% correct) than random. Moreover, when presented with four items, the distribution of the *K*-values of all participants indicated the discrete performance rather than a concentrated distribution range (Distribution of each *K*-value range: *k* < 1.5: 4; 1.5 < *k* < 2: 6; 2 < *k* < 2.5: 11; 2.5 < *k* < 3: 12; 3 < *k* < 3.5, 4; *k* > 3.5: 1). Thus, we propose that performance was sufficiently sensitive to the measure in our experiment. Gold et al. (2007) have suggested that the Change “Location” Task, in which participants are asked to point to the specific location of a color change, is a more sensitive measure. We will consider using this measure in future studies to increase result validity.

Interestingly, we found that the benefits of training were transferrable across tasks, enhancing performance in a verbal WM task for which the participants had received no training. These results suggest that FE plays an important central executive role in WM tasks. Owens et al. (2013) posited that capacity differences may be due to differences in inhibition. In a study with children diagnosed with ADHD, Cornoldi et al. (2001) found that memory deficits were not due to weaker storage ability, but rather to poor inhibition. Inhibition is a main function of the central executive system, which allows attentional resources to be allocated to relevant information and cognitive processes while suppressing irrelevant information. Although visual and verbal WM are two different subsystems of WM (i.e., the visuospatial sketchpad and the phonological loop), both are coordinated by the central executive system (Baddeley and Hitch, 1974). Hence, the current study supports the view that the central executive system plays a pivotal role in WM. However, we did not find significant correlations between verbal WM, Cowan’s *K*-values and FE. It is possible that as two distinct components of the WM system, VWM and verbal WM may not directly interact. As discussed above, the transfer ability of the benefits of training across tasks was due to the inhibitory ability of central executive functioning. The inhibition ability we trained here may be about the visual perceptual aspect, while the inhibitions in verbal WM focuses more on verbal aspect (Luo et al., 2003). That is the reason causing the loss of the direct correlation between the two types of WM.

The relationship between intelligence and WM has been debated. Using the RSPM test, Klingberg et al. (2002) found that WM training could increase intelligence scores in children with ADHD. Conversely, Westerberg and Klingberg (2007), similar to the results presented here, found no effect of WM training on RSPM scores. Indeed, many studies have reported no significant transfer of WM gains to intelligence (Morrison and Chein, 2011). While some studies have reported WM training effects on intelligence (Klingberg et al., 2002; Olesen et al., 2004; Jaeggi et al., 2008), a recent meta-analysis demonstrated that these studies were not appropriately powered. Conversely, many of the participants in the present study obtained close to maximal RSPM scores at the pre-training assessment and it is therefore possible that a ceiling effect prevented the observation of a training effect on intelligence. It may be that the effects of training may benefit intelligence only in individuals with some form of cognitive impairment. Moreover, the participants in the current study finished the RSPM in a relatively short period of time compared to other studies. However, given that test-taking time does not have a strong influence on RSPM score (i.e., a high correlation exits between scores on a speed vs. non-speed version), it is unlikely that the short amount of time taken for the RSPM test in the current study could explain the lack of WM training effects on intelligence.

While the participants in the present study were cognitively normal, the training model could be applied to improve capacity in patients who have some deficits in WMC, or other cognitive deficits that correlate with WM capacity. Jost et al. (2011) found that the decline in WM functions in older adults is partly due to a decrease in FE. In addition, filtering deficits have also been associated with Parkinson’s disease (Lee et al., 2010) and anxiety (Qi et al., 2014). Furthermore, CDA is altered in groups with known deficits in VWM, such as aged individuals (Jost et al., 2011; Sander et al., 2011) and patients with ADHD (Wiegand et al., 2016). The results from the present study suggest that training may help reduce the differences in VWM between groups with known filtering deficits and normal groups.

While the present study provides novel results on the benefit of training in WM, it is not without its limitations. First, the study lacks an appropriate control group, necessary to clearly interpret the effects of the training procedure. For example, the possibility that the observed training effect was achieved through expectancy effects, rather than the training its self cannot be ruled out. Morrison and Chein (2011) proposed that the selectivity or system activity of training transfer influences expectancy effects. Therefore, if the training effects seen in the current study were only related to expectancy effects, then a more ubiquitous transfer would have been predicted. In addition, there was a selective transfer of benefits from training on VWM tasks to verbal WM, but not fluid intelligence. These results also argue against expectancy confounds. Second, since there was no control group, the influence of time on task performance cannot be dismissed. However, there was no change in capacity or filtering ability 3 months following training, which suggests that time may not be a significant factor. Third, it may be argued that due to the method for separation between the low-capacity subjects and the high capacity subject, the results may have been due RTM. However, the RTM analysis suggests that the change in capacity was most likely not due to a simple RTM effect. In addition, if the improvement in scores resulted from an effect of RTM then the performance of the low-capacity group should regress toward the mean value (Nielsen et al., 2007). However, after training the low-capacity group performed significantly higher than the mean value, and was not significantly different from the high-capacity group. Moreover, there was no change in performance at the 3 month follow-up compared to the post-training assessment, indicating that the increase in low-capacity performance could not be attributed simply to an RTM effect. Lastly, participants in the current study performed better than participants in other studies, (Vogel et al., 2005; Lee et al., 2010; Owens et al., 2013). If RTM effect worked, then the performance in post and follow-up phase would be close to the mean value like others’ results. Future studies should contain a “control training” group whose experience is closely matched to the training group in order to more concretely rule out the above confounds.

## Ethics Statement

Capital Normal University Human Research Committee before participating in the experiments, all gave written informed consent, and were compensated for their time. We did not involve minors, persons with disabilities or endangered animal species in our study.

## Author Contributions

Conceived and designed the experiments: C-HL and C-YG. Performed the experiments: C-HL. Analyzed the data: C-HL and ZH. Wrote the paper: C-HL, XH, Y-JW, ZH, and C-YG.

## Conflict of Interest Statement

The authors declare that the research was conducted in the absence of any commercial or financial relationships that could be construed as a potential conflict of interest.
